# Recent Progress in Biomimetic Anisotropic Hydrogel Actuators

**DOI:** 10.1002/advs.201801584

**Published:** 2019-01-15

**Authors:** Xiaoxia Le, Wei Lu, Jiawei Zhang, Tao Chen

**Affiliations:** ^1^ Key Laboratory of Marine Materials and Related Technologies Zhejiang Key Laboratory of Marine Materials and Protective Technologies Ningbo Institute of Material Technology and Engineering Chinese Academy of Sciences Ningbo 315201 China; ^2^ College of Materials Science and Opto‐Electronic Technology University of Chinese Academy of Sciences 19A Yuquan Road Beijing 100049 China

**Keywords:** anisotropic structures, biomimetic actuation, hydrogel actuators, stimuli‐responsive hydrogels

## Abstract

Polymeric hydrogel actuators refer to intelligent stimuli‐responsive hydrogels that could reversibly deform upon the trigger of various external stimuli. They have thus aroused tremendous attention and shown promising applications in many fields including soft robots, artificial muscles, valves, and so on. After a brief introduction of the driving forces that contribute to the movement of living creatures, an overview of the design principles and development history of hydrogel actuators is provided, then the diverse anisotropic structures of hydrogel actuators are summarized, presenting the promising applications of hydrogel actuators, and highlighting the development of multifunctional hydrogel actuators. Finally, the existing challenges and future perspectives of this exciting field are discussed.

## 1 Introduction

**Figure 1:**
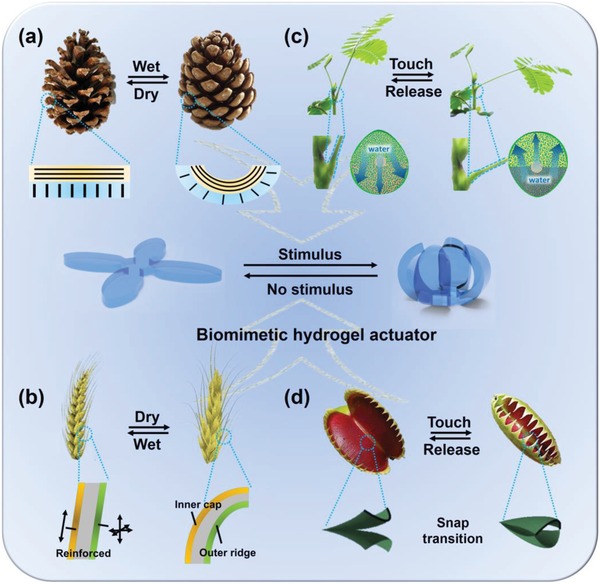
The design of hydrogel actuators is inspired by the movements of natural plants, such as a) the opening of a pinecone (adapted with permission.^1^ Copyright 2012, Annual Reviews), b) the bending of wheat awns upon humidity stimulus (adapted with permission.^2^ Copyright 2007, AAAS), c) the droop of Mimosa (adapted from with permission.^4^ Copyright 2018, Royal Society of Chemistry), and d) the closure of Venus flytrap triggered by touching (reproduced with permission.^5^ Copyright 2007, John Wiley and Sons).

Nature is a perpetual source to inspire the development of artificial intelligent materials that can adapt and actuate in response to external stimuli. In particular, living creatures from unicellular organisms to humans could generate movements upon environmental stimuli. The actions of animals are normally based on the contraction of certain muscles, while the motions of plants are mainly driven by water absorption or dehydration of cells. For instance, pinecone scales are formed by two kinds of tissues with different orientation of cellulose fibrils, the inhomogeneous local swelling/shrinking in humid environments will trigger the opening or closing of pinecone (**Figure** **1**a).^1^ The same mechanism was found to contribute to the bending of wheat awns.^2^ Different from pinecone that needs to exchange water with the environment, Mimosa^3^, ^4^ and Venus Flytrap^5^ could generate motions by a redistribution of water inside their tissues (Figure 1c,d).

As one of the most important intelligent materials, polymeric hydrogel actuators^6^, ^7^, ^8^, ^9^ could transfer various external stimuli such as heat,^10^, ^11^ pH,^12^, ^13^ light,^14^, ^15^ chemicals,^16^ electricity^17^, ^18^, ^19^ to controllable and reversible shape transformation. Moreover, compared with other polymeric actuators like liquid crystalline actuators,^20^, ^21^, ^22^ hydrogel actuators are soft and wet materials, just like the living organisms. Therefore, they have aroused tremendous attention and shown promising applications^23^, ^24^, ^25^, ^26^ as soft robots, artificial muscles, valves, and so on. It is worth noticing that the design principles of most hydrogel actuators come from natural plants.

Several years ago, a number of excellent reviews involving hydrogel actuators have been published,^9^, ^25^, ^27^, ^28^ and the general aspects of hydrogel actuators have been covered. However, with the rapid development of this field, especially in the last five years, we feel it is highly necessary and important to summarize the notable progress and discuss future perspectives of polymeric hydrogel actuators. In this review, the development and general design strategies of polymeric hydrogel actuators will be briefly described first, then the fabrication of different architectures will be introduced, followed by the promising applications of hydrogel actuators. Finally, the current challenges and future perspectives in this field will be discussed to provide a new developing direction and inspiration for future research.

## 2 General Consideration

The driving force of polymeric hydrogel actuators is uptake and release of water. If the hydrogel actuators have isotropic structures, only simple homogeneous swelling/shrinking could be achieved under uniform stimuli, which limits their further applications. With the development of hydrogel actuators, complex deformations/movements have been explored to expand their potential applications. The current investigated approaches to realize complex deformations/movements can be divided into two main categories: 1) imposing external nonuniform stimuli such as electric field or local light irradiation onto isotropic hydrogels^29^, ^30^, ^31^ and 2) preparation of internal anisotropic hydrogels.^32^ Because of the difficulty of applying nonuniform external stimuli precisely onto hydrogel actuators, the fabrication of anisotropic structures has become more and more popular to achieve complex deformations. These hydrogel actuators could provide various complex deformations/movements upon uniform stimuli due to the heterogeneous responsiveness of inhomogeneous properties. In this review, we will focus on hydrogel actuators with anisotropic structures.

## 3 Hydrogel Actuators with Anisotropic Structures

**Figure 2:**
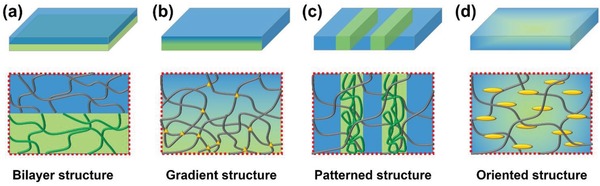
The cartoon figures of various anisotropic structures, including bilayer structure, gradient structure, patterned structure, and oriented structure.

It is a universal principle that the potential applications of materials are predominantly determined by properties, which are essentially determined by structures. Therefore, the design and fabrication of anisotropic structures is fundamental for the development of hydrogel actuators. Up to now, various inhomogeneous structures including bilayer structures,^33^, ^34^, ^35^ gradient structures,^36^, ^37^, ^38^ patterned structures,^39^, ^40^, ^41^, ^42^ oriented structures,^43^, ^44^, ^45^ and some others^46^, ^47^ have been explored (**Figure** **2**). In this section, we are going to discuss the diverse anisotropic structures of hydrogel actuators.

### 3.1 Bilayer Structures

Bilayer hydrogel actuators, consisting of two hydrogel sheets with different swelling rates or ratios, have been successfully developed to perform controllable deformations such as bending and bucking on the basis of asymmetrical responsive properties of the two parts. Several approaches including layer‐by‐layer polymerization, assembly of different hydrogels via reversible switches, such as host–guest interactions, and hydrogen bonding, have been explored to construct bilayer hydrogel actuators.

Layer‐by‐layer polymerization^26^, ^35^, ^48^, ^49^, ^50^, ^51^ is a widely used strategy for fabricating hydrogel actuators with bilayer structures. In the preparation process, the monomer solution of the second layer will slightly penetrate into the first layer, leading to the formation of an interpenetrating‐network at the interface, which will act as a junction layer to connect the two layers tightly. For instance, Chu and co‐workers have developed a series of poly(*N*‐isopropylacrylamide)(PNIPAM)–clay nanocomposite hydrogels as temperature‐controlled manipulators. Through tuning the thickness ratio of the two hydrogel layers with different clay contents, the thermoresponsive bending direction and degree of the hydrogel actuators could be adjusted.^49^ Employing a similar way, Yoon and co‐workers have reported a static‐motion bilayer hydrogel actuator, which is composed of PNIPAM‐graft‐methylcellulose (PNIPAM‐g‐MC) with a large thermal hysteresis as the active layer, and polyacrylamide (PAAm) as the passive layer. By integrating magnetic nanoparticles with photothermal effect, a bilayer actuator capable of static bending upon light irradiation can be realized.^50^

**Figure 3:**
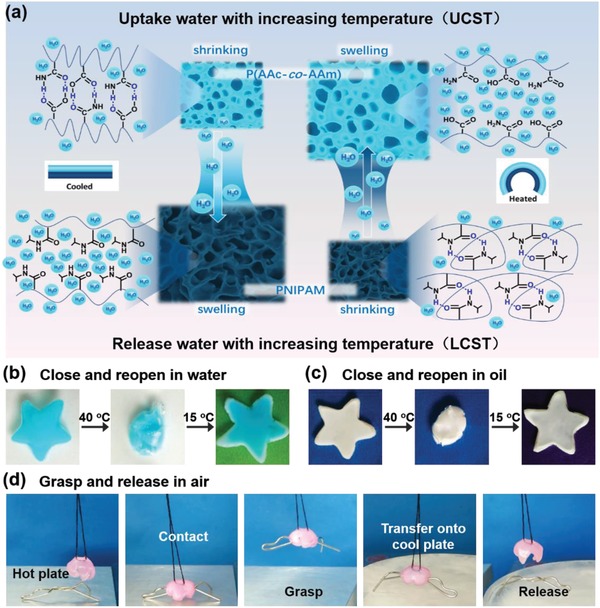
a) A bilayer hydrogel consisting of a PNIPAM layer and a P(AAc‐co‐AAm) layer is fabricated, the PNIPAM layer would release water with increasing temperature, while the P(AAc‐co‐AAm) layer would uptake water with increasing temperature, and the bilayer hydrogel would bend as a result. b) Closing and reopening of a hydrogel flower at 40 and 15 °C in water. c) Closing and reopening of a hydrogel flower at 40 and 15 °C in paraffin. d) The hydrogel gripper grasps a heated metal wire, lifts it up, and releases it when brought in contact with an ice‐cold surface. Adapted with permission.^4^ Copyright 2018, Royal Society of Chemistry.

In addition, hydrogel sheets with different functions can also be incorporated via the method of layer‐by‐layer polymerization. In our previous work, inspired by Mimosa with a reversible water transfer between two compartments of pulvinus,^4^ we have developed a thermoresponsive bilayer hydrogel actuator with internal water self‐circulation. PNIPAM hydrogel with a lower critical solution temperature (LCST) and poly(acrylic acid‐co‐acrylamide) (P(AAc‐co‐AAm)) hydrogel with an upper critical solution temperature (UCST) are combined. Upon the change of temperature, water molecules would migrate between the LCST layer and the UCST layer within the bilayer hydrogel (**Figure** **3**a). Therefore, the responsiveness of this hydrogel actuator is not induced by water exchange with external environment, and allows for actuation in water, oil, and even in air (Figure 3b–d). This water self‐circulation within the bilayer hydrogel offers a novel and smart strategy for actuators working in multienvironments.

**Figure 4:**
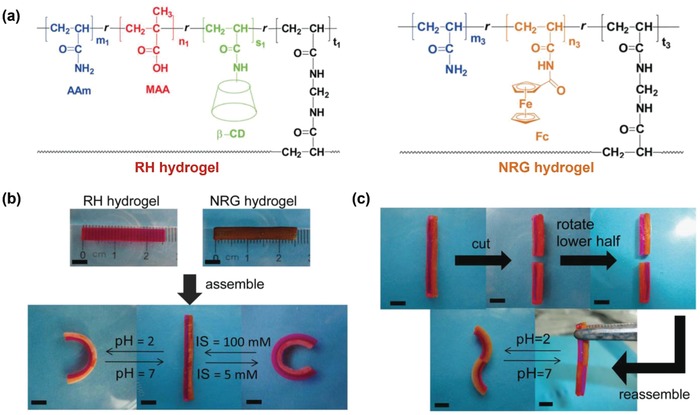
a) Chemical structures of RH and NRG hydrogels. b) Reversible bidirectional bending performance of a bilayer hydrogel assembled from the RH and NRG hydrogels. c) Enhanced responsive shape changing behavior of a bilayer hydrogel. Reproduced from with permission.^54^ Copyright 2014, John Wiley and Sons.

Because of the dynamic and reversible nature of molecular switches,^52^, ^53^ there is a great advantage to use them to create smart bilayer hydrogel actuators with the assembly of two homogenous hydrogel sheets.^35^, ^54^, ^55^, ^56^, ^57^ For example, Xie and co‐workers have unprecedentedly constructed hydrogel actuators through supramolecular building blocks assembly approach.^54^ First of all, they have fabricated responsive host hydrogel with β‐cyclodextrin moieties and carboxylic groups (RH hydrogel), and nonresponsive guest hydrogel with ferrocene groups (NRG hydrogel) (**Figure** **4**a). Taking advantage of the reversible host–guest interaction between β‐cyclodextrin and ferrocene, bilayer hydrogel actuator which could bend upon the change of pH and ionic strength has been assembled by the RH hydrogel and the NRG hydrogel (Figure 4b). Moreover, the bilayer hydrogel sheet could be cut into two halves and rejoined to provide more complex bending behavior (Figure 4c). In addition, using polymer chains containing adamantine guest groups as supramolecular glue, different hydrogel sheets bearing host groups can also be integrated.^56^ Compared with the layer‐by‐layer polymerization technique, assembly of different hydrogels via reversible switches has proved to be an effective and convenient way to fabricate bilayer hydrogel actuators.

Though hydrogel actuators with bilayer structures have been widely investigated, they could only perform shape deformations such as bending. In order to expand their potential applications, hydrogel actuators with other inhomogeneous structures have been developed.

### 3.2 Gradient Structures

Hydrogels with gradient structures, either gradient distribution of polymer chains or fillers, are efficient to produce complex shape deformations. Embedding stimuli‐responsive nanoparticles and utilizing the migration of nanoparticles under external electric or magnetic fields during the polymerization process provide an effective way to create hydrogel actuators with gradient structures. For example, Akashi and co‐workers have utilized the method of electrophoresis and subsequent photopolymerization to fabricate thermoresponsive PNIPAM hydrogels with either gradient silica nanoparticles or nanopores.^37^ The designed nanostructured gradient hydrogel would deform in response to the change of temperature due to the inherent bending character. Applying a similar strategy, Liu et al. have introduced magnetic nanoparticles (MNPs) into clay/PNIPAM nanocomposite hydrogels to achieve programmable responsive shaping behaviors.^44^ In this system, visible multidimensional gradients of MNPs in hydrogels can be realized by using different magnetic fields during in situ polymerization, leading to various “programmed” shape transformation behaviors.

**Figure 5:**
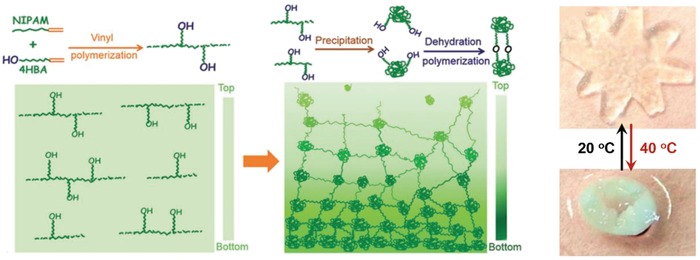
Scheme illustrating the hydrothermal synthesis of gradient porous hydrogel containing NIPAM and 4HBA. Reproduced with permission.^36^ Copyright 2015, John Wiley and Sons.

Besides the assistance of nanoparticles, hydrogel actuators with gradient structures can also be achieved by asymmetric distribution of polymer chains.^36^, ^38^ Recently, Chen and co‐workers have presented a gradient porous elastic hydrogel through a heterobifunctional cross‐linker enabled hydrothermal process.^36^ NIPAM was first polymerized with 4‐hydroxybutyl acrylate (4HBA) to form PNIPAM chains with hydroxyl groups, which can be precipitated and cross‐linked to generate the final gradient porous hydrogel (**Figure** **5**). Therefore, the hydrogel could exhibit rapid thermoresponsive folding/unfolding performance. Furthermore, by incorporating polypyrrole nanoparticles as photothermal transducers, laser‐driven programmable locomotion such as bending, curving, swimming can be achieved. The previous reported approach of fabricating gradient hydrogels usually requires special molecular design, or the assistance of external field during the polymerization process, and there is another simpler way for constructing hydrogel actuators with a gradient structure.^38^ Another example presented by our groups is that a uniform PAAm hydrogel was prepared as the primary network, then a secondary PAAc network was introduced through UV‐initiated polymerization. Because of limited penetration ability of UV light, a dense PAAc network would form on the top of the hydrogel, and a loose PAAc network would form on the bottom of the hydrogel, leading to an anisotropic structure. The obtained PAAm‐PAAc hydrogel could accomplish complex shape deformations upon the trigger of pH owing to the heterogeneous responsiveness of anisotropic structure.

### 3.3 Patterned Structures

**Figure 6:**
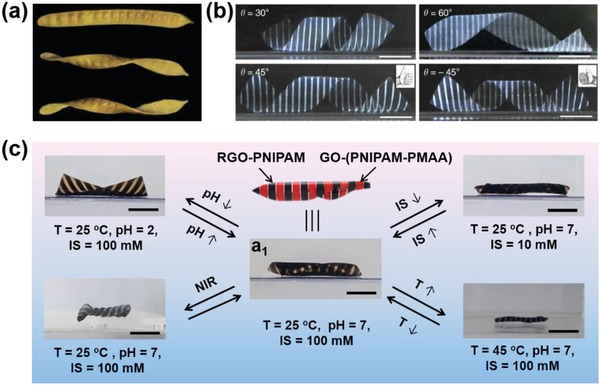
a) Stages of twisting of seed pods. Reproduced with permission.^58^ Copyright 2011, John Wiley and Sons. b) Images of the helices generated by anisotropic hydrogel sheets with photolithographed patterns. Reproduced with permission.^41^ Copyright 2013, Nature Publishing Group. c) Multiresponsive 3D complex deformations of anisotropic hydrogel under the stimulus of pH, IS, NIR light, and temperature, respectively. Reproduced with permission.^12^ Copyright 2016, John Wiley and Sons.

Nature has always been a remarkable supervisor of inspiring the development of hydrogel actuators. For instance, straight seed pods could twist into helical structures upon dehydration (**Figure** **6**a),^58^ which has encouraged the exploration of hydrogel actuators with anisotropic structures in plane to accomplish complex 3D structures. Photolithography is an effective approach to render hydrogel sheets with chemically distinct regions,^12^, ^40^, ^41^, ^42^ Nie and co‐workers have fabricated planar hydrogel sheet with small‐scale patterns of homopolymer and copolymer via photopatterning method, because of the differential in swelling/shrinkage of the patterned polymer components upon the change of pH, ionic strength, or temperature, the obtained hydrogel sheets could undergo preprogrammed large 3D shape transitions to acquire various different shapes including helical, conic shapes (Figure 6b).^41^ Applying photopatterning strategy, we have constructed multiresponsive anisotropic hydrogels with 3D complex deformations. Through local UV‐reduction of graphene oxide (GO) in the GO‐PNIPAM composite hydrogel sheet, followed by the introduction of a second poly(methylacrylic acid) network (PMAA) in the unreduced part, the obtained 3D hydrogel could provide pH‐, near‐infrared light (NIR)‐triggered, thermo‐, and ionic strength (IS)‐driven multiresponsive 3D complex deformations (Figure 6c).^12^ These results indicate photolithography is a convenient and universal approach to achieve anisotropic hydrogel actuators with multiresponsive 3D complex shape deformations, which will inspire the design and fabrication of more intelligent hydrogel actuators.

**Figure 7:**
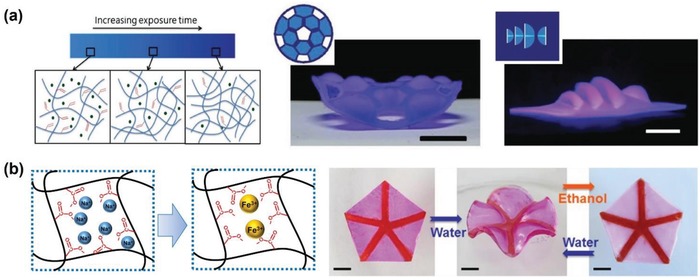
a) Illustration of the control of cross‐linking degree via digital light exposure, the obtained honeycomb‐shaped domes and 3D theater. Reproduced with permission.^39^ Copyright 2017, John Wiley and Sons. b) Cross‐linking between ferric ions and the carboxyl groups of hydrogel, the deformation of a hydrogel sheet with patterns of ferric ions. Adapted with permission.^60^ Copyright 2017, John Wiley and Sons.

Instead of introducing patterns of a second polymer network in a hydrogel sheet to generate stress, just tuning the cross‐linking density in specific region of the same hydrogel network could also accomplish complex transformations.^39^, ^59^, ^60^, ^61^ Applying only two photomasks, Hayward and co‐workers have created highly cross‐linked dots embedded in a lightly cross‐linked PNIPAM and benzophenone acrylamide copolymer films, and surfaces with constant Gaussian curvature or zero mean curvature have been successfully obtained.^59^ More recently, Xie and co‐workers^39^ have employed a computer‐controlled commercial projector to fabricate hydrogel networks with variable degree of cross‐linking densities. Compared with photomask method, the cross‐linking density at each pixel level could be precisely controlled by adjusting the light exposure time, which permits fine tuning of the gradient patterns, and sophisticated shapes such as 3D structure with honeycomb‐shaped domes and 3D theater have been constructed (**Figure** **7**a). Besides controlling the polymerization degree, the introduction of metal ions into hydrogel systems through either ionoprinting or ion inkjet printing is another effective method.^16^, ^60^, ^61^, ^62^, ^63^, ^64^, ^65^ Wang and co‐workers have shown that just using ferric ions to increase the cross‐linking density of poly(sodium acrylate) containing hydrogels via ion transfer printing technique, hydrogel sheets with designed patterns on one or both surfaces would be obtained, and the hydrogel sheets could thus perform complex deformations upon swelling/deswelling (Figure 7b).^60^ The above introduced approaches have offered various options to create patterned hydrogel actuators and realize versatile transformation behaviors, which will provide new opportunities in the designing and fabricating of intelligent soft materials for bioinspired applications.

### 3.4 Oriented Structures

**Figure 8:**
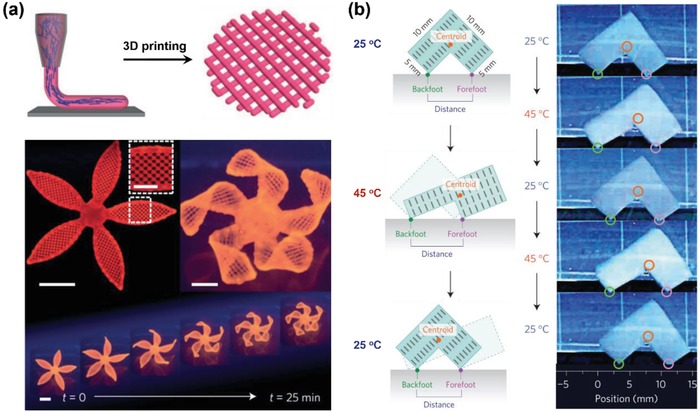
a) 3D printed hydrogel actuators with oriented cellulose nanofibrils. Reproduced with permission.^46^ Copyright 2016, Nature Publishing Group. b) Unidirectional procession of an L‐shaped PNIPAM‐based hydrogel actuator with cofacially aligned TiNS. Reproduced with permission.^43^ Copyright 2015, Nature Publishing Group.

It is well known that many plants including pinecones exhibit actuating performance on the basis of different local swelling behavior that arises from the directional orientation of cellulose fibrils. Inspired by that, polymeric hydrogel actuators with oriented nanofillers have been explored.^46^ Applying shear force on the monomer dispersion with nanofillers, the nanofillers will align to the shear direction, and anisotropic hydrogel with oriented structures could be obtained after polymerization. Lewis and co‐workers have utilized a viscoelastic solution that contains acrylamide monomer, photoinitiator, embedded stiff cellulose nanofibrils, and so on as ink, and the cellulose fibrils tend to orient to the shear force during the 3D printing procedure, leading to the asymmetric swelling behavior of the longitudinal direction and the transverse direction. They have printed various plant‐inspired architectures, and these structures could undergo shape change in water and achieve complex 3D morphologies (**Figure** **8**a). However, shear force cannot be homogeneously utilized for preparing thick samples,^32^ Aida and co‐workers have applied magnetic field as an alternative strategy to produce anisotropic hydrogel actuators with considerable thickness.^43^ Unilamellar titanate (IV) nanosheet (TiNS) will cofacially align in a magnetic field, and a thermoresponsive PNIPAM‐based hydrogel with oriented TiNS was developed, and the hydrogel could perform anisotropic deformations because of the expansion and contraction of the cofacial TiNS distance upon the trigger of temperature, which was caused by the changes of electrostatic repulsion between the cofacially oriented TiNS. By taking advantage of the asymmetric shape deformation, an L‐shaped hydrogel actuator with an oblique nanosheet conformation has been designed and could display unidirectional procession (Figure 8b). Except for the orientation of nanofiller, oriented void channels fabricated through directional freezing could also endow hydrogels with anisotropic structure.^66^, ^67^, ^68^

### 3.5 Other Anisotropic Structures

**Figure 9:**
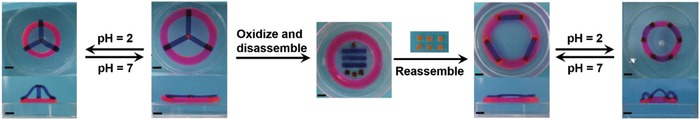
Building blocks hydrogel with shape deformation behavior. Reproduced with permission.^54^ Copyright 2014, John Wiley and Sons.

Besides bilayer structures, gradient structures, patterned structures, oriented structures, hydrogel actuators with other anisotropic structures have also been emerged to realize complex shape deformations.^35^, ^47^, ^54^, ^69^, ^70^ Inspired by building blocks assembly, Xie and co‐workers have demonstrated that utilizing robust yet reversible molecular switches as the chemical locking forces between hydrogel blocks not only could afford simple bilayer structures, but could also achieve 3D responsive architectures.^54^ It is worth noting that the 3D structures could be disassembled because of the reversible nature of molecular switches, and the hydrogel blocks could be reassembled into a new structure that could undergo 2D to 3D reversible shape transformation (**Figure** **9**). Employing the strong hydrogen bonding between polymers and clay nanosheets, Chu and co‐workers have built various assembled hydrogel architectures for achieving diverse shape deformations.^35^ In theory, the building blocks assembly approach has the potential to create unlimited anisotropic structures, and the actuating behaviors of the built structures would be significantly improved if hydrogel blocks with more sophisticated stimuli‐responsiveness are involved, therefore this strategy will certainly broaden the field of hydrogel actuators.

Moreover, with the development of 3D printing, stimuli‐responsive hydrogels with various shapes have been designed and constructed, which will create new opportunities for the further developing of polymeric hydrogel actuators.

## 4 Promising Applications of Hydrogel Actuators

As mentioned above, the potential applications of hydrogel actuators are closely related to their reversible shape transforming performances. Though still in the conceptual stage, hydrogel actuators have been designed as grippers,^12^, ^49^ walkers,^29^, ^71^ swimmers,^36^ artificial muscles,^72^, ^73^ valves,^74^, ^75^ and so on to realize corresponding functions such as grasping, transporting, and releasing objects, walking or swimming, lifting, and controlling the flow flux. Recently, the most popular applications of hydrogel actuators are as grippers, walkers, and swimmers. In this section, we will discuss the potential applications of anisotropic hydrogel actuators.

### 4.1 Manipulators or Grippers

**Figure 10:**
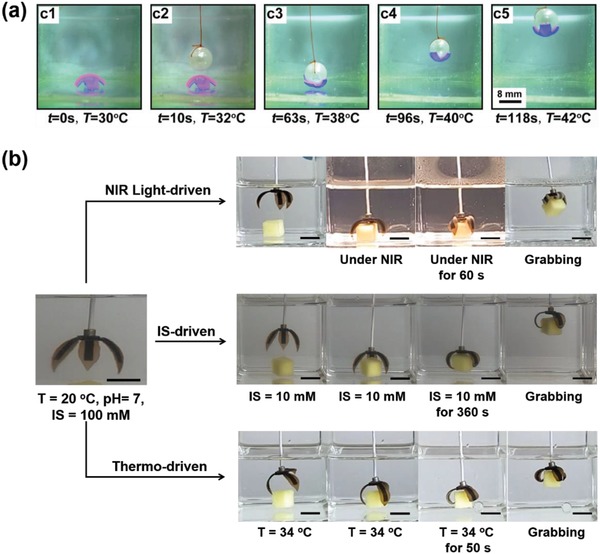
a) Transportation of a hydrogel gripper by a moving pearl. Reproduced with permission.^49^ Copyright 2015, John Wiley and Sons. b) The grabbing process of a multiresponsive hydrogel actuator under the stimuli of NIR, IS, and temperature. Reproduced with permission.^12^ Copyright 2016, John Wiley and Sons.

One of most promising applications of inhomogeneous hydrogel actuators is as manipulator or gripper with three or more arms, which can capture object when exposed to one stimulus and release it under another.^33^ Applied a bilayer PNIPAM–clay nanocomposite hydrogel with different content of clay, Chu and co‐workers have shown the transportation of the hydrogel sheet by a moving pearl. Because the shrinking ratio of the two layers is different upon the increasing of temperature, the cross‐shaped hydrogel sheet will bend toward the side with less clay, as a result, it will tightly wrap and move with the pearl which is manipulated by a string (**Figure** **10**a).^49^ Though many hydrogel grippers have been reported, most of the hydrogel grippers could only respond to one stimulus, and it is difficult to design manipulating devices with multiresponsive actions. Using the anisotropic PNIPAM‐PMAA hydrogel with local reduced GO, we have designed a multiresponsive soft gripper which can grasp an object quickly and firmly upon the trigger of several different stimuli including NIR light, temperature, and IS (Figure 10b).^12^ This work provides an effective approach to fabricate hydrogel manipulators that could work in many fields, which would shed light on the designing of intelligent hydrogel grippers for further applications.

### 4.2 Walkers and Swimmers

**Figure 11:**
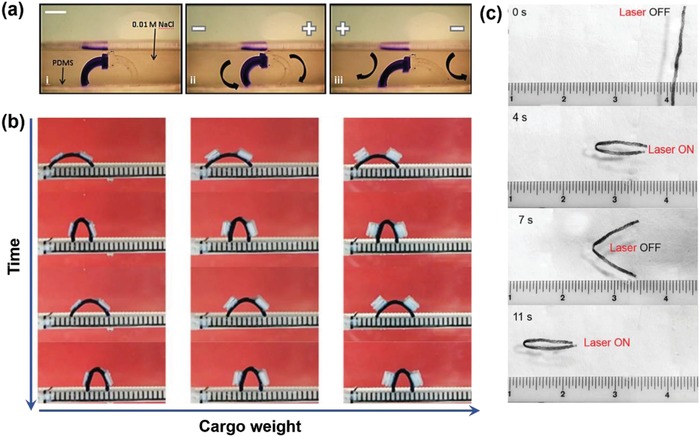
a) The walking performance of an electroactuated hydrogel walker. Reproduced with permission.^29^ Copyright 2014, Royal Society of Chemistry. b) Hydrogel walkers loaded with different weights. Reproduced with permission.^76^ Copyright 2015, Nature Publishing Group. c) Hydrogel swimmer driven by laser irradiation. Reproduced with permission.^36^ Copyright 2015, John Wiley and Sons.

Applying cyclical stimuli, bending actions can be transformed in walking or swimming performance.^71^ Velev and co‐workers have presented an electrodriven hydrogel walker with dual polyelectrolyte legs.^29^ The cationic and anionic hydrogel legs were made of acrylamide (AAm)/sodium acrylate and acrylamide/quaternized dimethylaminoethyl methacrylate, respectively. Through controlling the direction of the electric field, the hydrogel walker could march forward in dilute salt solutions on a flat substrate by using the two legs that deformed in opposite directions under electric field (**Figure** **11**a). Applying “on/off” electrotriggers, Chu and co‐workers have developed a hydrogel walker consisting of polyanionic poly(2‐acrylamido‐2‐methylpropanesulfonic acid‐co‐acrylamide) with gradient structures. The bending/stretching behaviors of the hydrogel walker could be well‐controlled via repeated electrical triggers, therefore one‐directional walking motion could be achieved on a rough surface based on the moving of the two legs. Moreover, the hydrogel walker also exhibits excellent walk performance even with very heavy cargo, and shows promising applications in transportation (Figure 11b).^76^ Most of the reported hydrogel walkers are promoted by electronic field, through designing PNIPAM‐based hydrogel with cofacially aligned TiNS nanosheets. Aida and co‐workers have constructed an L‐shaped hydrogel walk which could perform unidirectional procession under the trigger of cyclic temperature change (Figure 8b).^43^ Hydrogel actuators could also exhibit swim behavior under cyclic external stimuli. Chen and co‐workers have demonstrated an octopus‐like hydrogel swimmer fabricated by PNIPAM‐based gradient hydrogel with embedded polypyrrole nanoparticles.^36^ If the middle part of the hydrogel strip is irradiated by laser, the hydrogel strip would bent in the direction of the top surface and generated propulsive force, therefore the hydrogel strip would move forward (Figure 11c). During repeated laser irradiation, the hydrogel strip would swim in the aqueous solution.

### 4.3 Biomimetic Devices

**Figure 12:**
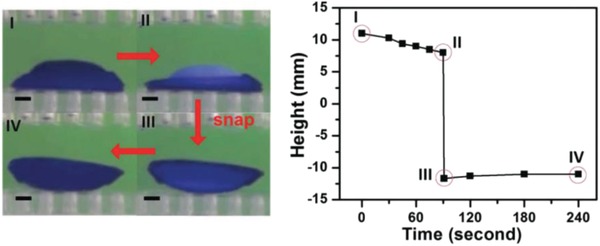
Reversible snapping hydrogel assembly and the corresponding height in the process of transformation. Reproduced with permission.^56^ Copyright 2016, Royal Society of Chemistry.

Since the design principles of most hydrogel actuators are derived from natural world, it is highly anticipated that hydrogel actuators could be applied to mimic the movement of living organisms. Inspired by the snapping transformation of Venus Flytrap, Xie and co‐workers have constructed heterogeneous hydrogel assemblies with an abrupt noncontinuous fashion (**Figure** **12**).^56^ With the aid of supramolecular glue, three kinds of hydrogel building blocks have been adhered to achieve snapping hydrogel assemblies with a bistable state. Through adjusting the stimuli of pH or temperature, the biomimetic Venus Flytrap could undergo reversible convex‐to‐concave transformation in a snapping style, which could provide unique advantages for hydrogel actuators.^5^ By imitating the water self‐circulation mechanism that contributes to the movement of Mimosa, we have developed artificial Mimosa leaves that could generate motions without water supply of external environment.^4^ These successful attempts may promote the design of novel smart biomimetic hydrogel devices.

### 4.4 Valves

**Figure 13:**
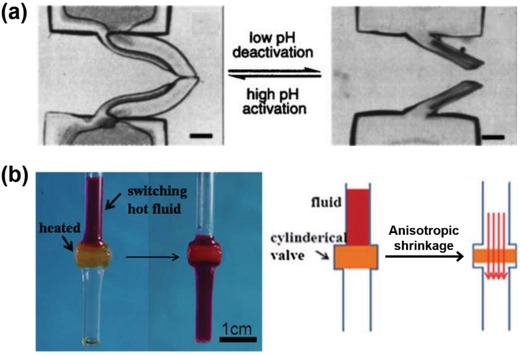
a) pH‐sensitive hydrogel bistrip valve. Reproduced with permission.^76^ Copyright 2015, American Institute of Physics. b) PNIPAM‐based hydrogel with anisotropic shrinkage behavior as a valve. Reproduced with permission.^75^ Copyright 2012, Royal Society of Chemistry.

The unique stimuli‐induced shape changing behavior makes hydrogel actuators promising candidates as valves in fluidic devices. Moore and co‐workers have presented a biomimetic hydrogel bistrip valve that could control the flow direction upon the trigger of local pH in microfluidic channels.^74^ As the hydrogel bistrip valve is comprised of a pH‐sensitive hydrogel strip and a pH‐unsensitive hydrogel strip, the volume and shape of the hydrogel valve would change reversibly by altering the local pH. In alkaline condition, the hydrogel bistrips could mimic anatomic venous valves both functionally and structurally due to unsymmetrically swelling of the two strips, enabling the fluid flow in one direction while preventing flow in the opposite direction (**Figure** **13**a). Through constructing anisotropic PNIPAM‐based hydrogel with an aligned porous polyethylene glycol (PEG) hydrogel matrix, Sun and co‐workers have demonstrated an efficient fluid regulation system with a centimeter scale hydrogel valve.^75^ At high temperature, the hydrogel valve would be activated because of the anisotropic shrinkage behavior, allowing the fluid to pass the valve (Figure 13b). Since hydrogel actuators could response to various external stimuli, hydrogel valves that are sensitive to many other stimuli could also be developed to meet practical applications in fluidic systems.

## 5 Multifunctional Hydrogel Actuators

Most of the hydrogel actuators only have shape deformation behaviors, in order to develop the potential applications of hydrogel actuators, recent attention has been paid to integrate some other functions such as fluorescence and shape memory into hydrogel actuators.

**Figure 14:**
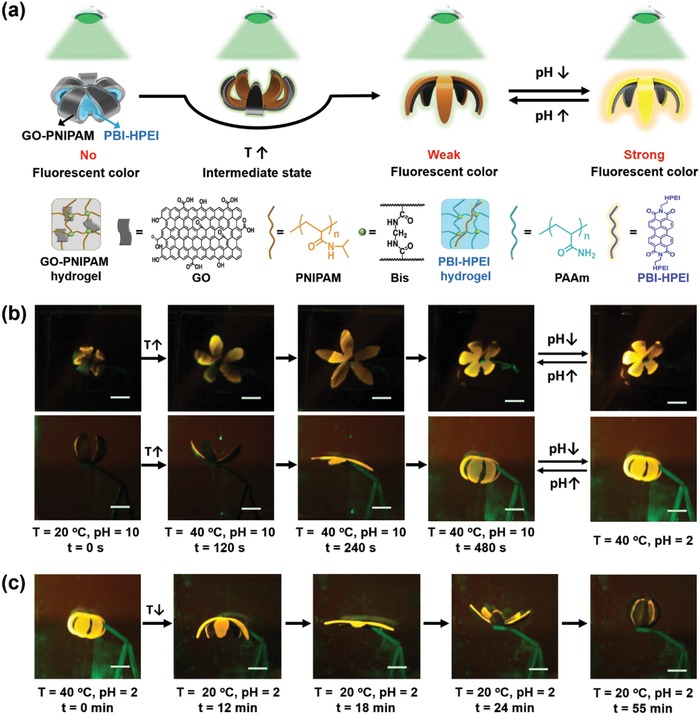
a) Illustration of the fabrication of bilayer hydrogel actuators with fluorescence color‐changing behaviors. b) The unfolding of the hydrogel actuator via increasing temperature and the change of fluorescence color upon the trigger of pH. c) The closing of the hydrogel actuator under the stimulus of temperature. Reproduced with permission.^57^ Copyright 2018, John Wiley and Sons.

Wei and co‐workers have presented a bilayer hydrogel actuator with fluorescent coumarin groups.^77^ Because of the anisotropic swelling of two layers, the bilayer hydrogel exhibits reversible shape deformation behaviors upon the stimulus of pH or temperature, accompanying with bright fluorescence of the entire hydrogel. This result suggests deformation and fluorescence performances could be simultaneously realized in one system. Inspired by some animals including chameleons, octopuses that not only could move but also can tune their body colors to camouflage in specific surroundings, we have designed a bilayer hydrogel actuator with on–off switchable fluorescent color‐changing functions.^57^ First of all, thermoresponsive GO‐PNIPAM hydrogel layer and pH‐responsive perylene bisimide‐modified hyperbranched polyethylenimine (PBI‐HPEI) hydrogel layer were prepared, respectively, then the two layers were bind together through host–guest interactions between β‐CD and adamantane to obtain 3D bilayer hydrogel actuators (**Figure** **14**a). At 20 °C, the fluorescent PBI‐HPEI hydrogel layer is wrapped by the GO‐PNIPAM hydrogel layer, the shape of the hydrogel actuator could deform via thermal stimulus caused by the shrinking of the outside GO‐PNIPAM layer, therefore the PBI‐HPEI hydrogel layer will be uncovered and exposed to the green light irradiation. Then the fluorescence intensity of the PBI‐HPEI hydrogel layer will be enhanced by changing the pH (Figure 14b). Moreover, both the actuating and fluorescence color‐changing performances are reversible (Figure 14c). This system has integrated fluorescence color‐changing functions with actuating performances, which may provide new insights into the designing of novel biomimetic hydrogel actuators with synergistic visually detecting functions.

**Figure 15:**
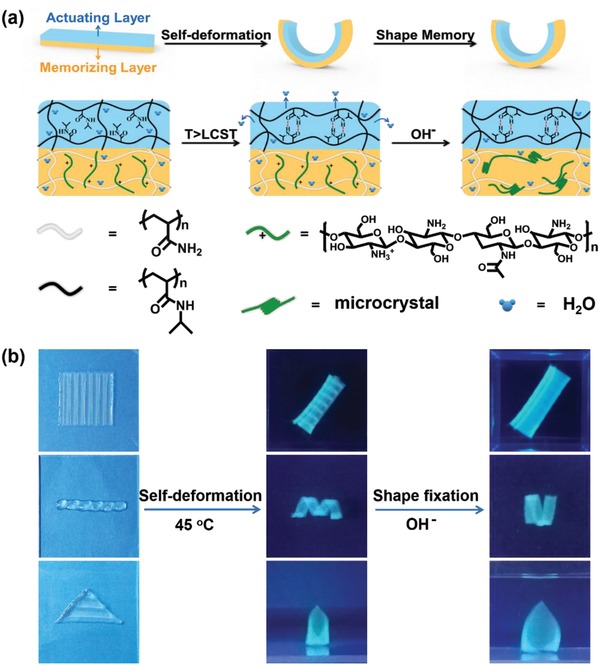
a) Illustration of hydrogel actuators with shape memory functions. b) The combination of actuating and shape deformation behaviors. Reproduced from with permission.^78^ Copyright 2018, Royal Society of Chemistry.

Including shape memory function, the deformed shape of a hydrogel actuator could also be fixed.^78^ Appling a thermoresponsive PNIPAM layer and pH‐responsive PAAm‐chitosan (PAAm‐CS) layer, we have presented bilayer hydrogel actuators with shape memory performances. When the temperature was increased, the hydrogel actuator would deform due to the shrinkage of PNIPAM layer, then the deformed shape could be fixed by the chitosan microcrystalline via the change of pH (**Figure** **15**a). If the PNIPAM actuating layer was deliberately fabricated as patterns on the top of PAAm‐CS memorizing layer, complex shape transformation would occur and be stabilized by the microcrystallization of chitosan in alkaline solution (Figure 15b). This strategy may inspire the design of advanced hydrogel actuators.

## 6 Conclusions and Outlooks

In the present review, we have presented the recent progress in the field of anisotropic polymeric hydrogel as biomimetic soft actuators. It is clear that through constructing bilayer, gradient, patterned, oriented, as well as other anisotropic structures, various deformations including bending, twisting, and 3D complex shape transformations could be achieved, leading to potential applications as grippers, walkers, swimmers, biomimetic devices, or valves. Moreover, some other functions such as fluorescence and shape memory could be integrated into hydrogel actuators, multifunctional hydrogel actuators have thus been developed to expand the potential applications of hydrogel actuators. However, in spite of the promentioned attractive achievements of hydrogel actuators, there remain challenges and opportunities in this field.

First of all, precise control of the shape deformation performance needs to be realized. New strategies such as digital printing and 3D printing have been explored to construct prescribed anisotropic structures, which provide exciting opportunities to accomplish better control of the actuating behaviors. Moreover, multistimuli responsiveness hydrogel actuators need further development to achieve programmed multistep deformation behaviors.

Second, though many efforts have been devoted to the preparation of multifunctional hydrogel actuators in the past few years, some other functions such as self‐healing are suggested to be incorporated to broaden to potential applications of hydrogel actuators.

Third, most of the applications of hydrogel actuators are still in their conceptual stage, the practical applications of hydrogel actuators should be explored and demonstrated in the future. In practical applications, hydrogel actuators have to be applicable in real world. To achieve this destination, they normally need to be strong enough and durable, however, the mechanical performances and environmental resistances are usually neglected in most of reported hydrogel actuators. To this end, hydrogel actuators with excellent mechanical properties should be constructed.

Last but not least, hydrogel actuators with quick responsiveness are also needed to be constructed. Through reducing the size, hydrogel actuators in microscale or nanoscale exhibit not only quick responsiveness but also excellent stability compared with traditional polymeric micelles or vesicles,^79^, ^80^ showing promising applications in the biological field. Moreover, for practical applications, the generated power, and the energy transfer efficiency between external energy and mechanical energy needs to be concerned.

The abovementioned challenges in the field of hydrogel actuators need mutual efforts from interdisciplinary experts with different backgrounds. We believe that there will be vigorous development in biomimetic anisotropic polymeric hydrogel actuators in the near future.

## Conflict of Interest

The authors declare no conflict of interest.
